# Biogenic Synthesis of NiO Nanoparticles Using *Areca catechu* Leaf Extract and Their Antidiabetic and Cytotoxic Effects

**DOI:** 10.3390/molecules26092448

**Published:** 2021-04-22

**Authors:** Shwetha U R, Rajith Kumar C R, Kiran M S, Virupaxappa S. Betageri, Latha M S, Ravindra Veerapur, Ghada Lamraoui, Abdulaziz A. Al-Kheraif, Abdallah M. Elgorban, Asad Syed, Chandan Shivamallu, Shiva Prasad Kollur

**Affiliations:** 1 Research Centre, Department of Chemistry, GM Institute of Technology, Davangere 577 006, Karnataka, India; shwethaur@gmail.com (S.U.R.); rajithcr91@gmail.com (R.K.C.R.); kiranmsafare@gmail.com (K.M.S.); 2 Department of Chemistry, R L Science Institute, Belagavi 590 001, Karnataka, India; lathamschem97@gmail.com; 3 Department of Metallurgy and Materials Engineering, Malawi Institute of Technology, Malawi University of Science and Technology, Limbe P.O. Box 5916, Malawi; rveerapur@must.ac.mw; 4 Faculty of Nature and Life Sciences, Earth and Universe Sciences, University of Tlemcen, Tlemcen 13000, Algeria; lamraouig@gmail.com; 5 Dental Biomaterials Research Chair, Dental Health Department, College of Applied Medical Sciences, King Saud University, P.O. Box 10219, Riyadh 11433, Saudi Arabia; aalkhuraif@ksu.edu.sa; 6 Department of Botany and Microbiology, College of Science, King Saud University, P.O. Box 2455, Riyadh 11451, Saudi Arabia; aelgorban@ksu.edu.sa (A.M.E.); assyed@ksu.edu.sa (A.S.); 7 Department of Biotechnology and Bioinformatics, School of Life Sciences, JSS Academy of Higher Education and Research, Mysuru 570 015, Karnataka, India; chandans@jssuni.edu.in; 8 Department of Sciences, Amrita School of Arts and Sciences, Amrita Vishwa Vidyapeetham, Mysuru Campus, Mysuru 570 026, Karnataka, India

**Keywords:** *Areca catechu*, NiO NPs, TEM, antidiabetic activity, anticancer potential

## Abstract

Nanoworld is an attractive sphere with the potential to explore novel nanomaterials with valuable applications in medicinal science. Herein, we report an efficient and ecofriendly approach for the synthesis of Nickel oxide nanoparticles (NiO NPs) via a solution combustion method using *Areca catechu* leaf extract. As-prepared NiO NPs were characterized using various analytical tools such as powder X-ray diffraction (XRD), scanning electron microscopy (SEM), transmission electron microscopy (TEM), and UV-Visible spectroscopy (UV-Vis). XRD analysis illustrates that synthesized NiO NPs are hexagonal structured crystallites with an average size of 5.46 nm and a hexagonal-shaped morphology with slight agglomeration. The morphology, size, and shape of the obtained material was further confirmed using SEM and TEM analysis. In addition, as-prepared NiO NPs have shown potential antidiabetic and anticancer properties. Our results suggest that the inhibition of α-amylase enzyme with IC 50 value 268.13 µg/mL may be one of the feasible ways through which the NiO NPs exert their hypoglycemic effect. Furthermore, cytotoxic activity performed using NiO NPs exhibited against human lung cancer cell line (A549) proved that the prepared NiO NPs have significant anticancer activity with 93.349 μg/mL at 50% inhibition concentration. The biological assay results revealed that NiO NPs exhibited significant cytotoxicity against human lung cancer cell line (A549) in a dose-dependent manner from 0–100 μg/mL, showing considerable cell viability. Further, the systematic approach deliberates the NiO NPs as a function of phenolic extracts of *A. catechu* with vast potential for many biological and biomedical applications.

## 1. Introduction

For the past few years, nanotechnology has acquired marvelous impetus by creating new scientific ideas in this rapidly growing technological era [1,2]. Nanomaterials have revealed many technological insights with their tremendous applications and specific properties [3,4]. Surface morphology, characteristic size, and shape are key features for nanomaterials, which make them highly attractive and more reactive for researchers [5,6]. Biologically fabricated nanoparticles with their immense applications in various fields are growing continuously through the collaboration of different natural science sectors. The world of nanotechnology may furnish a novel resource for the evaluation and development of safer, newer, and effective drug formulations in the treatment of infectious diseases [7].

Recently, the interest in synthesizing metal oxide nanoparticles has increasingly been employed in various fields due to their potential applications in memory storage devices, photocatalytic sensors, magnetic resonance imaging, drug delivery, catalysis, and biomedicine [8]. Nanoparticles exhibits cytotoxic activity due to their higher adsorption ability over bulk materials [9]. Hence, they are used to treat various tumor and cancer cells [10]. Nickel oxide is a p-type semiconductor metal oxide possessing a band gap from 3.6 to 4.0 eV that has great importance and has received enormous consideration in research owing to its peculiar properties like large surface area, high chemical stability, good electronic conductivity, and super conductance characteristics [11,12]. Its ecofriendly nature and high reactivity makes it a potential candidate for applications in the field of magnetism, electronics, energy technology gas sensors, electrochemical super capacitors, catalysis, battery cathodes, magnetic materials, fuel cells, optical fibers, and biomedicines [13,14]. Moreover, NiO nanostructures have motivated young researchers due to their easy availability with low cost, quantum size confinement, and surface-to-volume effect [15,16]. NiO NPs are synthesized by different physical and chemical methods, namely, Sol-gel, hydrothermal, precipitation, solvothermal, etc. However, the biogenic synthesis approach has drawn the attention of researchers due to its biocompatibility and ecofriendly process, which involves green synthetic routes that are less toxic. Exploiting the potential of medicinal plants is one of the green synthesis routes, which includes algae, microorganisms, plants, etc., and is significant because the current therapeutic approaches have toxicity problems and microbial multidrug resistance issues. Metal nanoparticles have received great attention across the globe, so, in this study, we discuss and focus on metallic nanoparticles obtained by green synthesis using medicinal plants. We also discuss medicinal properties like antidiabetic and anticancer activities of synthesized nanoparticles. The biomolecules, secondary metabolites, and coenzymes present in the plants help with the easy reduction of metal ions to nanoparticles. Such nanoparticles are considered as potential antioxidants and promising candidates in cancer treatment. Thus, the synthesis of ecofriendly nanoparticles from combustion solutions is one of the simplest and easiest synthetic approaches towards uniform mixing of plant extract with precursor/oxidizing agents [17].

Plants are known for their medicinal values in terms easier availability and large number of biologically active components. *A. catechu* is one of the known fruit plants belonging to the Palmaceae family and is cultivated in most Asian countries [18]. Medicinal properties of this plant’s extracts are due to the presence of various phytochemicals that are present in the different parts of the plant [19]. Perusal of the literature shows that *Areca* leaves possess more bioactive molecules, namely, arecoline, arecolidine, arecaidine, guvacoline, guvacine, and isoguvacine. Use of plant extracts for the synthesis of nanoparticles is desirable due to the various plant metabolites like polyphenols, alkaloids, phenolic acids, and terpenoids, which play a major role in the bioreduction of metal ions, yielding nanoparticles. Plant act as bioreactors in the binding and reduction of metal ions, thereby influencing the formation of nanoparticles.

In recent years, solution combustion synthesis is emerging as one of the efficient methods to produce nanomaterials with a controlled size and shape. It is also used as a rapid heating method for metal oxides synthesis. Beyond rapid heating, this green synthesis method gives good product yield in less time when compared to other conventional methods. The present study sheds light on the synthesis of highly efficient, cost effective, nanosized NiO nanoparticles by using the solution combustion synthesis method. Solution combustion synthesis is a green, efficient, simple, fast, and high-yield method. The novelty of the study is the use of Areca catechu leaf extract as a reducing and stabilizing agent for NiO nanoparticles synthesis. Temperature plays a pivotal role here. The solution combustion reaction depends on various process parameters, and it plays a significant role in phase formation, phase stability, and physical characteristics. The reaction temperature is a crucial parameter in the synthesis of materials. The released heat of the combustion reaction fulfils the energy requirement for the formation of oxides. The presence of phytochemical constituents in the plant extract; concentration of plant extract; and reaction conditions like temperature, reducing agent concentration, reaction time, and size of nanoparticles all influence the stability of NiO NPs [20].

The size and morphology of the nanoparticles play a significant role in developing the chemical and physical properties and largely influence their existing applications. Therefore, much effort was dedicated to the fabrication of NiO NPs with different sizes and morphologies. The decrease in dimension leads to an increase in the surface area and this enhances the biological properties.

In the current study, *A. catechu* leaf extract is used as a reducing and stabilizing agent to synthesize NiO NPs. Prepared nanoparticles were characterized using XRD, SEM with EDAX, and HR-TEM. Furthermore, we investigated the cytotoxicity of NiO NPs by examining cell viability and antidiabetic activity. This study provides detailed information about the cytotoxic effects of as-prepared NiO NPs against human lung cancer cells and offers a sound basis for the clarification of its toxicity mechanisms. 

## 2. Materials and Methods

All the chemicals were analytical grade, procured from SD Fine and Himedia Laboratory Pvt. Ltd., India, and used without further purification. The morphology of as-prepared NiO NPs was observed by Transmission Electron Microscopy (TEM-1011, JEOL, Tokyo, Japan). SEM with Energy dispersive X-ray Analysis was utilized to evaluate the elemental study (Hitachi S3400n, Tokyo, Japan). X-ray diffraction examination of NiO NPs was done on a PANalytical X’Pert-PRO (Rigaku Smart Lab). UV-Visible spectrophotometer (Shimadzu UV-2450, Kyoto, Japan) was used to record electronic absorption spectra. 

### 2.1. Preparation of Areca Catechu Leaf Extract and Synthesis of NiO NPs

*Areca Catechu* leaves were collected from the local areas near Davanagere. Freshly collected leaves were washed with double distilled water, dried, and grinded well to get fine powder. To prepare the leaf extract, 10 g of *A. catechu* leaf powder was boiled in 100 mL distilled water for 30 min at 60 °C. Further, the extract was filtered and dried under vacuum using a rotary evaporator.

The solution combustion method was used to synthesis NiO NPs. In a typical experiment, 10 mL of *A. catechu* leaf extract and 1 g of nickel nitrate hexahydrate Ni(NO_3_)_2_6H_2_O) were taken in a silica crucible and placed in a preheated muffle furnace maintained at 500 °C. An exothermic, vigorous reaction leads to the formation of fine, black colored NiO NPs. The obtained product was kept in an airtight container for further analysis [21].

### 2.2. Antidiabetic Activity: Inhibition of Alpha Amylase Enzyme Assay 

Pancreatic α-amylase belongs to the class of α-1,4-gluconohydrolases and is one of the important target enzymes for the conventional treatment of diabetes. It catalyzes the initial step in hydrolysis of starch to maltose and maltotriose, which are then acted upon by α-glucosidases, broken down into glucose, and enter the blood stream. Naturally available α-amylase inhibitors from medicinally important plants are shown to be very effective in managing postprandial hyperglycemia, which is a major concern in type 2 diabetes [22].

In a fresh tube, 1 mL of phosphate buffered saline (PBS) solution was mixed with 0.5 mL of different concentrations (100, 200, 300, 400, and 500 µg/mL) of samples or the standard solution, then 200 µL of 0.5 mg/mL α-amylase was added followed by 200 µL of 5 mg/mL starch solution and incubated for 10 min at room temperature. Control was taken as starch with amylase and without α-amylase. Then, the reaction mixture was stopped by adding 400 µL of Dextrose normal saline (DNS) solution, followed by heating the mixture in a boiling water bath for 5 min, then cooling. The reaction without *A. catechu* leaf extract was used as a control. Metformin was used as a standard drug [23]. Inhibition of enzyme activity was calculated using the following formula:

% Inhibition of enzyme activity = Abs sample − Abs control / Abs sample × 100.(1)

### 2.3. Anticancer Activity: Cytotoxicity Assay of NiO NPs

The cytotoxicity assay of biosynthesized NiO NPs was performed against human lung cancer cell line (A549). The cell lines were cultivated in Dulbecco’s Modified Eagle’s Medium (DMEM) with fetal bovine serum, with antibiotics as supplements. Temperature was maintained around 37 °C with humidified 5% CO_2_ atmosphere for about 24 h. The cells were seeded in 96-well plates at a density 25×10^3^ cells/well. Cytotoxicity of biosynthesized NiO NPs was studied using 3-(4,5-dimethylthiazol-2-yl)-2,5-diphenyl tetrazolium bromide (MTT) assay. Here, human cancer cell lines were treated with different concentrations of NiO NPs (20 to 100 mg/mL from stock). The plate was removed from the incubator and the drug-containing media was aspirated. A total of 100 μL of medium containing 10% MTT reagent was then added to each well to get a final concentration of 0.5 mg/mL, and the plate was incubated at 37 °C and 5% CO_2_ atmosphere for 3 h. The culture medium was removed completely without disturbing the crystals formed. Then, 100 μL of solubilization solution (DMSO) was added and the plate was gently shaken in a gyratory shaker to solubilize the formed formazan [22].

The absorbance was measured using a microplate reader at a wavelength of 570 nm and also at 630 nm. The percentage growth inhibition was calculated, after subtracting the background, the blank, and the concentration of test drug needed to inhibit cell growth by 50% (IC_50_). Yellow color MTT dye turning to purple color due to the reduction of formazon crystals in the presence of cytotoxic activity shows in the mitochondrial succinate dehydrogenase enzyme in viable cells. The amount of 50% inhibition concentration was obtained by plotting the dose-dependent curve [24].

## 3. Results and Discussion

### 3.1. XRD Analysis

**Figure 1:**
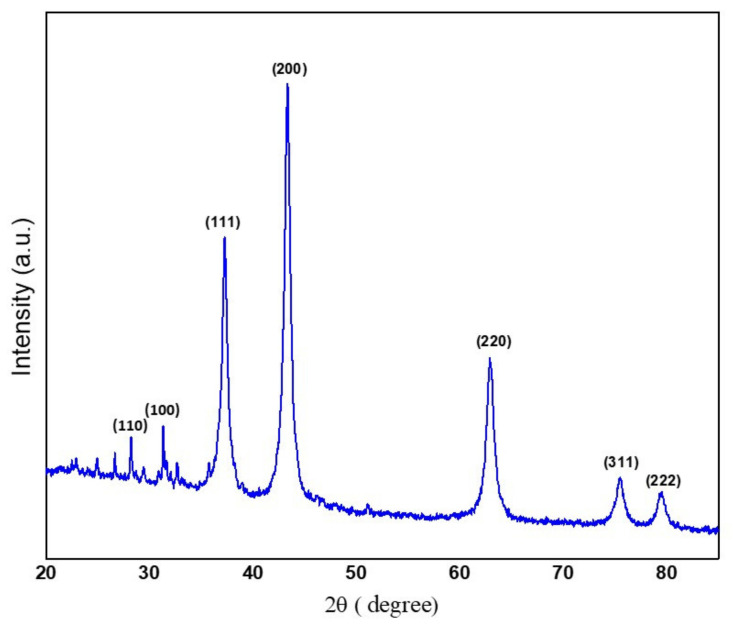
X-ray diffraction patterns revealing the crystal planes of as-prepared NiO NPs.

**Figure 2:**
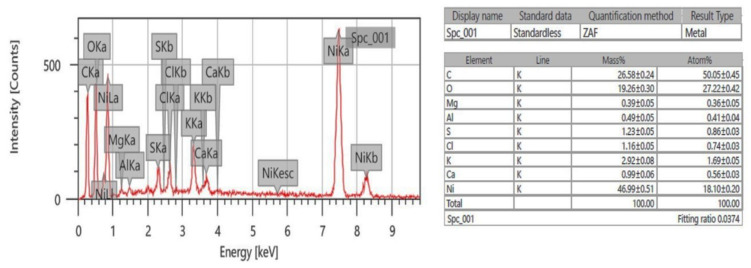
Energy-dispersive X-ray (EDAX) spectra depicting the chemical composition of the synthesized NiO NPs.

The XRD pattern of green synthesized NiO NPs from *Areca catechu* leaf extract show strong diffraction peaks at 37.23°, 43.29°, 62.88°, and 75.45°, which are assigned to the crystal planes (111), (200), (220), and (311), respectively, as shown in Figure 1, and are further well matched with JCPDS card no. 4-835. These planes indicate the formation of FCC cubical structure for NiO NPs. Further, no impurities were observed, which suggests a high purity of monophasic NiO NPs. The average crystalline size found to be 5.63 nm, calculated by the Debye–Scherer formula [25]. Moreover, the EDAX spectra of nanoparticles displayed the peaks of Ni and O, as seen in Figure 2, suggesting the chemical nature of the prepared material. The obtained profile of the synthesized nanoparticles confirmed the presence of nickel and oxygen in the nanoparticles.

### 3.2. UV-Visible Spectral Analysis

**Figure 3:**
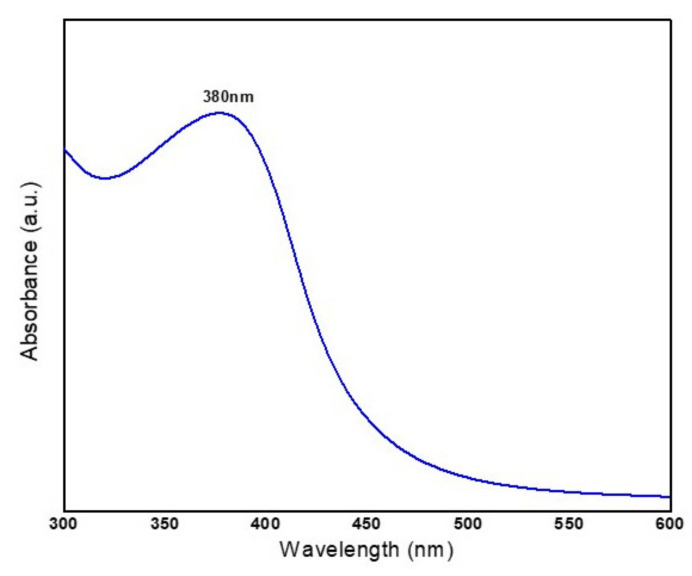
UV-Visible Spectrum of as-prepared NiO NPs.

It is clear from the UV-Visible spectrum of as-prepared NiO NPs (Figure 3) that the maximum absorption band observed at 380 nm reveals the formation of pure NiO NPs. This absorption in the UV region can be attributed to the electronic transition from the valence band to the conduction band in the NiO semiconducting nanocrystals. 

### 3.3. SEM Analysis

**Figure 4:**
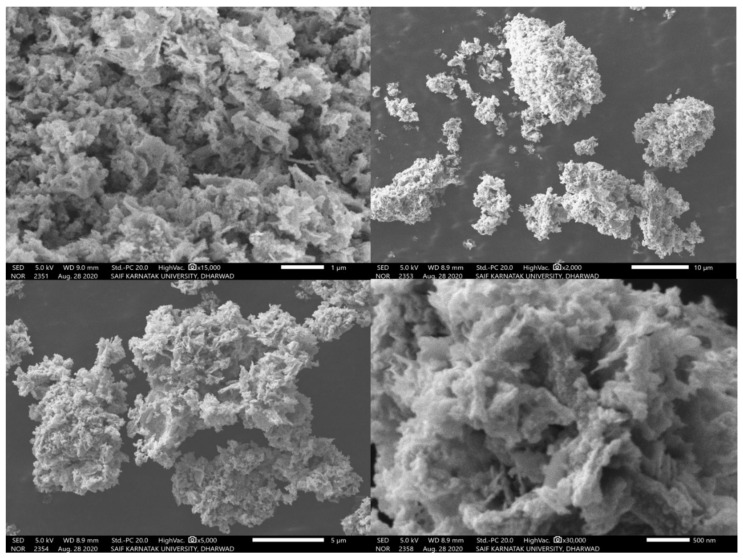
SEM images showing the morphology of as-prepared NiO NPs with different magnifications.

The surface morphological features of synthesized NiO NPs was studied using scanning electron microscope (SEM). In Figure 4, the SEM micrographs show the agglomeration with irregularly shaped nanoparticles. It can also be seen that the particles have a hexagonal shape with some degree of agglomerations, which may be attributed to the fact that NiO nanoparticles have high surface energy and high surface tension.

### 3.4. TEM Analysis

**Figure 5:**
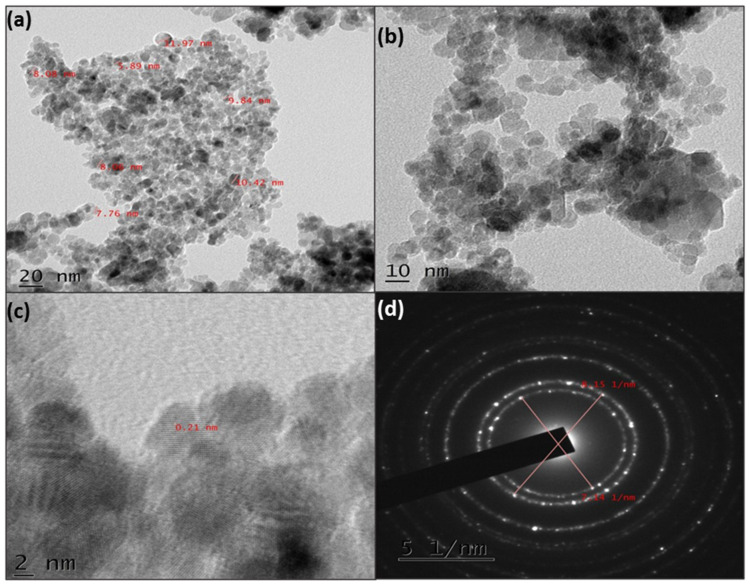
(**a**,**b**) TEM images, (**c**) HR-TEM image, and (**d**) SAED of as-prepared NiO NPs.

The formation of NiO NPs was perceived in the TEM images (Figure 5), which specifies the particle size within the range of 5 to 15 nm. Further, this supports the average crystal size from the XRD pattern. Figure 5b,c represent the HR-TEM micrographs that show particles in the hexagonal and rhombohedral shapes with an interplanar spacing of 0.21 nm. The selected area electron diffraction (SAED) pattern depicted in Figure 5d indicates the presence of the (111), (200), and (220) planes of the synthesized rhombohedral NiO NPs.

### 3.5. Antidiabetic Studies 

#### In Vitro Alpha Amylase Inhibition Method 

In our digestive system, pancreatic α-amylase is a key enzyme that catalyzes the initial step in the hydrolysis of starch. It is the main source of glucose in the diet. α-amylase inhibitors are those that inhibit the amylase activity that results in the delay of carbohydrate digestion and prolongs overall carbohydrate digestion time, causing a reduction in the rate of glucose absorption and consequently reducing the postprandial plasma glucose rise.

**Figure 6:**
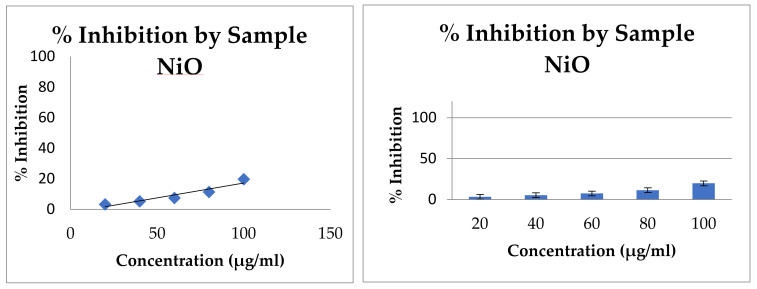
Antidiabetic potential of as-prepared NiO NPs showing inhibition of α-amylase activity at different concentrations.

The α-amylase inhibitor effectiveness of NiO NPs was compared with standard drug Metformin. The values were presented with graphical representation of the same in Figure 6. Alpha amylase is an enzyme that hydrolyses α-bonds of large α-linked polysaccharides such as glycogen and starch to yield glucose and maltose. α-amylase inhibitors bind to α-bond of polysaccharide and prevent the breakdown of polysaccharides in mono- and disaccharide. Standard drug Metformin showed inhibitory effects on the α-amylase activity with an IC_50_ value of 232.12 μg/mL. Prepared NiO NPs from Areca leaves exhibited α-amylase inhibitory activity with an IC_50_ value of 268.13 μg/mL. As a result, as-synthesized NiO NPs showed significant antidiabetic activity compared to Metformin. Moreover, drugs that inhibit carbohydrate hydrolyzing enzymes have been demonstrated to decrease postprandial hyperglycemia and improve impaired glucose metabolism without promoting insulin secretion of noninsulin-dependent diabetic patients. The results of in vitro studies showed that NiO NPs inhibits α-amylase activity [26].

As-prepared NiO NPs showed a percentage inhibition of 3.35 and 19.77 at 20 μg/mL and 100 μg/mL, respectively. The IC_50_ value of the extract was found to be 268.13 μg/mL, whereas the IC_50_ value of metformin was observed to be 232.12 μg/mL (Table 1). The concentration-based inhibition was noticed and the same has been depicted in Figure 6. Metformin is a standard antidiabetic drug and is competitively and reversibly inhibiting the pancreatic α-amylase. The retardation of glucose diffusion is also due to the inhibition of α-amylase, thereby limiting the release of glucose from the starch. The inhibition of α-amylase activity by medicinal plants might be attributed to several possible factors such as fiber concentration; the presence of inhibitors on fibers; and the encapsulation of starch and enzymes by the fibers present in the sample, thereby reducing accessibility of starch to the enzyme and direct adsorption of the enzyme on fibers, leading to decreased amylase activity. Thus, the inhibition of α-amylase activity is important to control postprandial hyperglycemia in the treatment of diabetes [27].

### 3.6. Cytotoxicity Studies 

**Figure 7:**
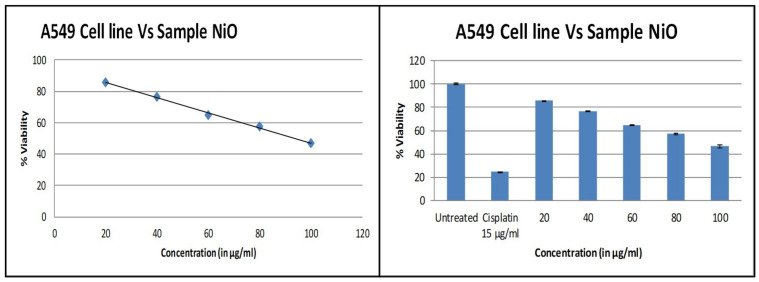
Graph representing the screening of anticancer activity with respect to the standard control for different concentrations of synthesized NiO NPs.

**Figure 8:**
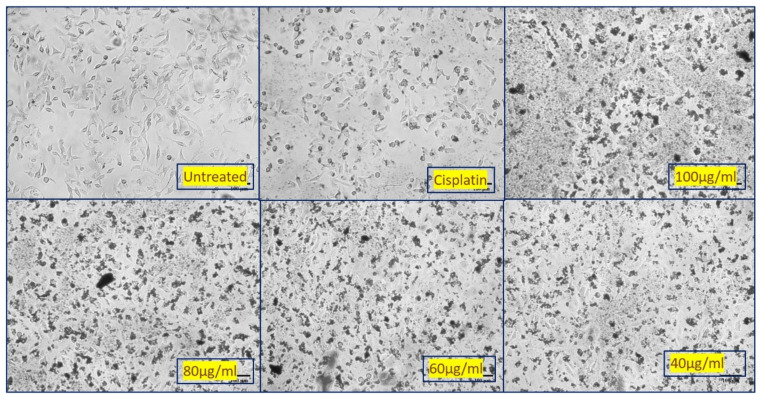
The viability assay of cytotoxicity of NiO NPs against cancer cell line (A549) treated with different concentrations of NiO NPs.

The evaluation of cytotoxicity of biosynthesized NiO NPs against A549 cell line cancer cells was measured based on cellular reduction of MTT during in vitro analysis. The as-prepared NiO NPs was screened against cell lines with the respective positive control Cisplatin, as shown in the Figure 7. NiO NPs treatment enhanced the cell death and also inhibited A549 cell population in a concentration-dependent manner. After treatment with different concentrations (20, 40, 60, 80, and 100 µg/mL), the plating efficiency of A549 cells declines, as proved by the reduction in the number of cancer cells formed. Exposure of various concentrations NiO NPs shows a decline in cell survival and plating efficiency. When compared with regular cisplatin, minimum inhibition was observed at 20 µg/mL and maximum at 100 µg/mL. The viability assay of cytotoxicity of NiO NPs against the cancer cell line is shown in Figure 8. Further, the IC_50_ density was found to be 93.349 µg/mL. The healthy and rapidly growing cells exhibit high rates of MTT reduction to formazan while the dead or inactive cells fail to do so. Viability in the MTT assay is connected linearly with enzyme activity and indirectly to the number of viable cells. The decrease in cell viability with the increasing concentration of NiO NPs shows significant cytotoxicity to accumulate in the internal cells and higher stress, ultimately leading to apoptosis [28,29].

## 4. Conclusions

In summary, we have reported the synthesis of NiO NPs by an ecofriendly approach via solution combustion method using the *Areca catechu* leaf extract. Areca is the important plant in Asia both in an agricultural role and as a traditional medicine. Preliminary phytochemicals like phenolic compounds, alkaloids, glycosides, and tannins are well-reported in literature. The X-ray diffractogram revealed the formation of hexagonal NiO NPs with a well crystalline nature and a very fine crystallite size of 5.63 nm. Further, the morphological characteristics determined by SEM and TEM analysis disclosed a size and shape of as-prepared nanostructures. Further, the antidiabetic activity of as-prepared NiO NPs was carried out using glucose uptake by yeast cell and α-amylase inhibition, which demonstrated significant antidiabetic activity. In addition, the prepared material showed potential anticancer activity against human lung cancer cell lines. The chemical constituents of areca plant had proven diverse pharmacological actions and were used as antidiabetic and anticancer agents. Overall, the present study clearly indicated that biosynthesized NiO NPs from *Areca catechu* leaves are a promising avenue for the prevention of diabetes and cancer diseases.

## Figures and Tables

**Table 1:** Antidiabetic activity of NiO NPs by α-amylase (pancreatic) inhibition assay by DNS method.

Sl. No	Concentrationsµg/mL	% Inhibition by Sample NiO NPs	% Inhibition by Standard DrugMetformin
1	20	3.35088	5.08616
2	40	5.39944	8.87984
3	60	7.45572	13.34370
4	80	11.44088	16.51507
5	100	19.77022	22.59454

## Data Availability

Data is contained within the article.
